# Current and Emerging Therapies for HER2-Positive Women With Metastatic Breast Cancer

**Published:** 2017-03-01

**Authors:** Karlyn Porter, Margaret Quinn Rosenzweig

**Affiliations:** 1 Magee-Womens Breast Cancer Program, University of Pittsburgh Cancer Institute, Pittsburgh, Pennsylvania;; 2 University of Pittsburgh School of Nursing, Department of Acute and Tertiary Care, Pittsburgh, Pennsylvania

## Abstract

**CASE STUDY**

DE, a 31-year-old premenopausal woman with a nonsignificant medical history, noticed a right breast mass after playing basketball in September 2011. She initially attributed the mass to slight trauma, but after 2 weeks, she realized the mass was increasing in size. Her primary care physician ordered a bilateral screening mammogram and ultrasound.

Mammography revealed no evidence of malignancy in the left breast. In the right breast, at the 7 o’clock position, a loose cluster of faint calcifications spanned a 2.2-cm area. Ultrasound confirmed an irregular hypoechoic mass in the right breast measuring 3.5 × 2.7 × 2.8 cm. Ultrasound of the right axilla identified two enlarged right axillary lymph nodes. Ultrasound core-needle biopsy of the suspicious right breast mass confirmed invasive ductal carcinoma, nuclear grade 2, Ki67 index of 55%, estrogen receptor–positive (H score of 180), progesterone receptor–positive (H score of 135), HER2-positive (3+ on immunohistochemistry). Utilizing the TNM (tumor, node, metastasis) staging system, she was clinically staged with a stage IIB (cT2, cN1, M0) invasive breast tumor.

The computerized axial tomography (CT) scan of the chest, abdomen, and pelvis demonstrated the known right breast mass and two enlarged right axillary lymph nodes; however, no metastatic disease was noted. Nuclear bone scan revealed no bone metastases.

Her medical oncologist recommended she receive neoadjuvant chemotherapy. The patient was treated with 6 cycles of neoadjuvant docetaxel at 75 mg/m², carboplatin at an AUC (area under the curve) of 6, and trastuzumab (Herceptin) at 6 mg/kg (TCH), which she tolerated well.

She then underwent a right segmental mastectomy with axillary lymph node dissection and was found to have a residual 1.0-cm invasive ductal carcinoma, representing a 60% tumor volume reduction. None of 13 axillary lymph nodes were positive for disease. Pathologic staging confirmed a stage IA (ypT1, ypN0, M0) tumor.

DE completed 33 fractions of radiation therapy to the right breast. She initiated endocrine therapy with tamoxifen at 20 mg daily and received 1 year of maintenance trastuzumab (6 mg/kg). Due to vaginal discharge and weight gain, endocrine therapy was switched from tamoxifen to toremifene (Fareston), which she tolerated relatively well. She continued routine follow-up, with no evidence of disease.

In September 2014, DE presented to her primary care physician complaining of left hip pain. Magnetic resonance imaging (MRI) of the left hip revealed T2 hyperintense masses within the right anterior superior iliac crest, right sacrum, and left iliac body consistent with skeletal metastases. She was referred back to her medical oncologist, and per National Comprehensive Cancer Network (NCCN) guidelines, a biopsy of the suspicious lesion was obtained. The bone biopsy of the lytic lesion was consistent with metastatic breast cancer, which was estrogen receptor–positive, progesterone receptor–positive, and HER2-positive (3+ on immunohistochemistry).

Restaging CT scan of the chest, abdomen, and pelvis revealed new 4- to 6-mm pulmonary nodules, hilar and mediastinal lymphadenopathy, new liver lesions, and bone lesions. Nuclear bone scan confirmed multiple bone metastases of the right and left iliac bones and sternum. Complete blood cell count with differential and complete metabolic panel were within normal ranges. The CA 27-29 tumor marker for breast cancer was elevated at 495 U/mL (normal range, < 37 U/mL).

DE was understandably devastated by the new diagnosis. She questioned how the treatment plan was to be established. Her medical oncologist struck a somewhat optimistic tone. He explained that metastatic breast cancer was not yet considered to be curable, but periods of disease stability and chronicity were possible. He explained that the HER2 positivity was perhaps the most important factor in delineating her treatment options. He told her that current treatment options were numerous and increasing in number.

A crucial step in developing a treatment plan for patients with metastatic breast cancer involves repeating hormonal studies and validating the HER2 status on the biopsied metastatic lesion. Testing validation of HER2 is extremely important, as approximately 20% of all breast cancers express HER2 protein, and there can be differences between primary and metastatic presentations (Schott, 2017). Testing for HER2 is performed using a validated immunohistochemistry (IHC) or in situ hybridization (ISH)/fluorescent in situ hybridization (FISH) assay.

## TYPES OF HER2 TESTING

Immunohistochemistry is the combination of histologic, biochemical, and immunologic techniques used to identify specific tissues components by the interaction of target antigens with specific antibodies. As a result, the distribution and localization of targeted cellular components can be observed (Chen, Cho, & Yang, 2010).

The overexpression of HER2 protein product is noted by IHC and is interpreted by a pathologist. When IHC is 0 or 1+, HER2 is negative. If IHC is 2+, the result is equivocal, thus requiring automatic testing with ISH or acquisition of a new specimen and repeat HER2 testing using IHC. An IHC of 3+ is HER2-positive (National Comprehensive Cancer Network [NCCN], 2016).

In situ hybridization is a technique that allows the visualization and localization of targeted nucleic acid sequence within a tissue sample by using a reporter molecule (National Center for Biotechnology Information [NCBI] Probe, 2014). The ISH is the ratio between HER2 and chromosome 17 enumeration probe (CEP17). In situ hybridization is positive when the HER2/CEP17 ratio is greater than or equal to 2.0, despite the average HER2 copy number signals/cells. In situ hybridization is also positive regardless of the HER2/CEP17 ratio when the average HER2 copy number is greater than or equal to 6.0 signals/cells. In situ hybridization is negative if the HER2/CEP17 ratio is less than 2.0 and the average HER2 copy number is less than 4.0 signals/cell. In situ hybridization is equivocal when the HER2/CEP17 ratio is less than 2.0 and the average copy number is greater than or equal 4.0 signals/cell but less than 6.0 signals/cell. When it is equivocal, reflex testing should be conducted to verify HER2 status (NCCN, 2016). Now that DE has confirmed HER2 metastatic breast cancer, her physician is able to choose from several different treatment options.

## HER2-POSITIVE TREATMENTS FOR METASTATIC DISEASE

In addition to systemic chemotherapy, DE’s treatment team can utilize a group of targeted agents to treat her HER2-positive disease. Multiple combination treatment regimens are available and vary depending on hormonal status, metastatic disease involving visceral vs. bone-only disease, and previous exposure to HER2-targeted therapy. Since DE is a young female with metastatic visceral disease and no comorbidities, her physician can choose from several treatment regimens.

Recommendations state that women with hormone-negative or -refractory, HER2-positive, newly diagnosed metastatic breast cancer should be treated with a combination regimen of trastuzumab (Herceptin), pertuzumab (Perjeta), and a taxane (paclitaxel or docetaxel; Schott, 2017). Trastuzumab and pertuzumab are both tyrosine kinase inhibitors; however, they have different mechanisms of actions. Trastuzumab is a monoclonal antibody that attaches to the extracellular domain of the HER2 protein, thus promoting antibody-dependent cellular cytotoxicity by inhibiting cellular proliferation that causes overexpression of the HER2 protein (Schott, 2017).

Pertuzumab is also a monoclonal antibody that is administered with trastuzumab to target the HER2 protein. Specifically, pertuzumab attaches extracellularly to the arm of the HER2 molecule, thus promoting apoptosis by halting cell signaling and growth (Schott, 2017). Studies have shown that adding pertuzumab to trastuzumab and docetaxel improves overall response and progression-free survival rates as compared with trastuzumab and docetaxel (Baselga et al., 2012; Schott, 2017).

If a patient cannot tolerate pertuzumab, the NCCN recommends trastuzumab plus other cytotoxic agents such as anthracyclines, taxanes, working anti-metabolites, and other microtubule inhibitors. Examples of chemotherapy combinations include trastuzumab plus pacilataxel with or without carboplatin; trastuzumab plus docetaxel; trastuzumab plus vinorelbine; and trastuzumab plus capecitabine (NCCN, 2016). Due to cardiac toxicity, it is imperative that trastuzumab and anthracycline be used cautiously and that the appropriate cardiac monitoring is performed.

Another promising treatment option for individuals with visceral or symptomatic HER2-positive metastatic breast cancer is ado-trastuzumab emtansine (Kadcyla; formerly known as T-DM1), confirmed through the international MARIANNE trial. Ado-trastuzumab emtansine is a HER2 antibody-drug conjugate with a microtubule inhibitor (DM1) and thioether, which results in apoptosis and cell-cycle arrest.

The MARIANNE trial examined the efficacy of ado-trastuzumab emtansine as a first-line treatment option. The MARIANNE trial enrolled 1,095 patients with progressive/recurrent locally advanced breast cancer or previously untreated metastatic breast cancer with at least a 6-month treatment-free interval from neoadjuvant or adjuvant treatment with a taxane or vinca alkaloid. Ado-trastuzumab emtansine was compared with standard anti-HER2 therapy through three arms: trastuzumab plus docetaxel or paclitaxel (physician’s choice; trastuzumab/taxane); ado-trastuzumab emtansine plus placebo; and ado-trastuzumab emtansine plus pertuzumab.

Overall there was no significant difference among progression-free survival between trastuzumab and ado-trastuzumab emtansine as a first-line agent (Ellis et al., 2015; Schott, 2017). In February 2013, the US Food and Drug Administration (FDA) approved the use of ado-trastuzumab emtansine as single-agent treatment for patients with HER2-positive metastatic breast cancer that was previously treated separately or in combination with trastuzumab and a taxane. There is no FDA indication for use of ado-trastuzumab emtansine in individuals with HER2 de novo metastatic breast cancer (patients presenting with distant metastases at the time of diagnosis; National Cancer Institute [NCI], 2013).

Lapatinib (Tykerb) is another HER2-targeted therapy that can be used to treat metastatic breast cancer. Due to an increase in incident adverse events, lapatinib is typically administered as a later line of therapy. Lapatinib inhibits the signaling pathway of HER2 as it is a tyrosine kinase inhibitor against epidermal growth factor receptor 1 (EGFR1) and HER2.

The National Cancer Institute of Canada Clinical trial group MA.31 trial showed a significantly shorter progression-free survival between paclitaxel plus trastuzumab vs. lapatinib plus paclitaxel. Also, studies have revealed there was a difference in overall survival between paclitaxel plus trastuzumab vs. lapatinib plus paclitaxel. Toxicity was also higher with lapatinib, particularly diarrhea, rash, and anorexia. Similarly, febrile neutropenia was also noted more often in patients taking lapatinib (Gelmon et al., 2012; Schott, 2017). A benefit of lapatinib is that it is an oral drug, so it is ideal for patients who insist on oral therapies.

Lapatinib can also be combined with capecitabine as a treatment option for individuals whose disease progresses on trastuzumab. The combination of capecitabine and lapatinib revealed a significant benefit in the time to disease progression (median, 6 vs. 4 months). A comparison of lapatinib alone vs with trastuzumab showed that individuals receiving combination therapy had an improvement in progression-free survival (median, 11 vs. 8 weeks). Also, patients enrolled on the combination-therapy arm with lapatinib plus trastuzumab demonstrated a benefit, with improvement in overall survival compared with individuals receiving monotherapy with lapatinib alone (median, 14 vs. 10 months; Blackwell et al., 2010, 2012; Schott, 2017).

Ultimately, DE’s physician decided to treat her with ado-trastuzumab emtansine at 3.6 mg/kg, given every 3 weeks, due to her previous treatment with a taxane and trastuzumab, ado-trastuzumab emtansine’s promising anti-HER2 activity, minimal side-effect profile, and lack of alopecia with this agent compared with other systematic cytotoxic agents. Currently, DE is clinically stable. She experiences intermittent diarrhea, which is controlled with loperamide, and occasional pruritus of her hands. She is scheduled for scans in 3 months. If her disease progresses through the current treatment options available, she could possibly enroll in a clinical trial exploring new HER2-positive metastatic breast cancer drugs.

## NEWER HER2-POSITIVE AGENTS

Several new and exciting treatment options for HER2-positive metastatic breast cancer are currently being tested in clinical trials. The first one is neratinib, an oral agent used to treat previously treated or resistant HER2-positive metastatic breast cancer. Neratinib is an irreversible tyrosine kinase inhibitor of HER2 and EGFR. Preliminary phase I data show promising antitumor activity and time of progression-free survival. Current ongoing clinical trials are examining the use of neratinib with paclitaxel, trastuzumab, and vinorelbine in individuals who have trastuzumab-refractory disease (Canonici et al., 2013; Markman, 2015).

Another novel treatment in development is poziotinib, a pan-HER2 tyrosine kinase inhibitor. A phase II trial is being conducted to investigate poziotinib as a potential salvage treatment option for patients who fail to respond to a taxane plus anthracycline (Park, 2016).

The development and utilization of HER2 therapies have been life-changing for patients such as DE and many others with HER2-positive breast cancer. With appropriate attention to testing, clinical guidelines, and new treatment options, DE can continue to benefit from HER2 treatment options.
